# Supplementary material for: Modeling glioblastoma heterogeneity as a dynamic network of cell states
Source: Mol Syst Biol. 2021 Sep 16;17(9):e10105. doi: 10.15252/msb.202010105 (PMC8444284; doi:10.15252/msb.202010105)
Supplement: Supplementary file 5 — Source Data for Figure 3 [file MSB-17-e10105-s001.zip › Figure3A_sourcedata/GSEA_3065/nosigdb_state1.GseaPreranked.1622718409535/LARSSON_STATE_4.html]

Details for gene set LARSSON\_STATE\_4[GSEA]

|  || Dataset | state1 |
| Phenotype | NoPhenotypeAvailable |
| Upregulated in class | na\_pos |
| GeneSet | LARSSON\_STATE\_4 |
| Enrichment Score (ES) | 0.601897 |
| Normalized Enrichment Score (NES) | 2.1688693 |
| Nominal p-value | 0.0 |
| FDR q-value | 0.0 |
| FWER p-Value | 0.0 |
Table: GSEA Results Summary

  

Fig 1: Enrichment plot: LARSSON\_STATE\_4      
 Profile of the Running ES Score & Positions of GeneSet Members on the Rank Ordered List

  

| PROBE | GENE SYMBOL | GENE\_TITLE | RANK IN GENE LIST | RANK METRIC SCORE | RUNNING ES | CORE ENRICHMENT || 1 | UBE2S |  |  | 19 | 0.634 | 0.0479 | Yes |
| 2 | PTTG1 |  |  | 40 | 0.520 | 0.0867 | Yes |
| 3 | TUBA1B |  |  | 119 | 0.347 | 0.1060 | Yes |
| 4 | UBE2C |  |  | 125 | 0.340 | 0.1322 | Yes |
| 5 | H2AFZ |  |  | 153 | 0.321 | 0.1547 | Yes |
| 6 | RANBP1 |  |  | 154 | 0.320 | 0.1799 | Yes |
| 7 | LSM4 |  |  | 162 | 0.316 | 0.2040 | Yes |
| 8 | HMGN2 |  |  | 164 | 0.313 | 0.2285 | Yes |
| 9 | CDKN3 |  |  | 178 | 0.307 | 0.2513 | Yes |
| 10 | RAN |  |  | 194 | 0.297 | 0.2731 | Yes |
| 11 | CKS2 |  |  | 195 | 0.297 | 0.2965 | Yes |
| 12 | BIRC5 |  |  | 202 | 0.295 | 0.3190 | Yes |
| 13 | DTYMK |  |  | 205 | 0.292 | 0.3418 | Yes |
| 14 | SIVA1 |  |  | 252 | 0.276 | 0.3588 | Yes |
| 15 | DEK |  |  | 296 | 0.261 | 0.3749 | Yes |
| 16 | CKS1B |  |  | 346 | 0.242 | 0.3889 | Yes |
| 17 | LSM3 |  |  | 361 | 0.237 | 0.4061 | Yes |
| 18 | PTMA |  |  | 366 | 0.236 | 0.4243 | Yes |
| 19 | ALYREF |  |  | 371 | 0.234 | 0.4423 | Yes |
| 20 | TUBB |  |  | 398 | 0.226 | 0.4574 | Yes |
| 21 | PHF19 |  |  | 415 | 0.223 | 0.4733 | Yes |
| 22 | LSM5 |  |  | 448 | 0.216 | 0.4870 | Yes |
| 23 | H2AFV |  |  | 538 | 0.198 | 0.4935 | Yes |
| 24 | TPX2 |  |  | 556 | 0.194 | 0.5070 | Yes |
| 25 | CCNB1 |  |  | 583 | 0.189 | 0.5192 | Yes |
| 26 | ANP32B |  |  | 622 | 0.183 | 0.5296 | Yes |
| 27 | SNRPD1 |  |  | 647 | 0.179 | 0.5413 | Yes |
| 28 | STMN1 |  |  | 763 | 0.163 | 0.5424 | Yes |
| 29 | CENPN |  |  | 764 | 0.163 | 0.5552 | Yes |
| 30 | HMGB1 |  |  | 995 | 0.134 | 0.5422 | Yes |
| 31 | RPA3 |  |  | 1039 | 0.129 | 0.5480 | Yes |
| 32 | TK1 |  |  | 1056 | 0.127 | 0.5563 | Yes |
| 33 | RFC2 |  |  | 1094 | 0.123 | 0.5622 | Yes |
| 34 | NUDT1 |  |  | 1144 | 0.118 | 0.5665 | Yes |
| 35 | CENPU |  |  | 1249 | 0.108 | 0.5644 | Yes |
| 36 | KIF4A |  |  | 1303 | 0.103 | 0.5671 | Yes |
| 37 | MAD2L1 |  |  | 1394 | 0.095 | 0.5654 | Yes |
| 38 | DHFR |  |  | 1413 | 0.094 | 0.5710 | Yes |
| 39 | UBE2T |  |  | 1440 | 0.093 | 0.5756 | Yes |
| 40 | H2AFX |  |  | 1479 | 0.090 | 0.5788 | Yes |
| 41 | TYMS |  |  | 1501 | 0.089 | 0.5837 | Yes |
| 42 | HNRNPD |  |  | 1509 | 0.088 | 0.5899 | Yes |
| 43 | NUCKS1 |  |  | 1540 | 0.087 | 0.5936 | Yes |
| 44 | HMGB2 |  |  | 1570 | 0.085 | 0.5973 | Yes |
| 45 | HIST1H1E |  |  | 1608 | 0.082 | 0.6000 | Yes |
| 46 | PBK |  |  | 1651 | 0.079 | 0.6019 | Yes |
| 47 | NUSAP1 |  |  | 1736 | 0.073 | 0.5991 | No |
| 48 | PIMREG |  |  | 1777 | 0.071 | 0.6005 | No |
| 49 | ZWINT |  |  | 1923 | 0.064 | 0.5907 | No |
| 50 | GMNN |  |  | 2097 | 0.055 | 0.5774 | No |
| 51 | CENPH |  |  | 2215 | 0.051 | 0.5694 | No |
| 52 | SMC4 |  |  | 2308 | 0.047 | 0.5637 | No |
| 53 | CENPM |  |  | 2348 | 0.045 | 0.5632 | No |
| 54 | MYBL2 |  |  | 2549 | 0.038 | 0.5458 | No |
| 55 | GINS2 |  |  | 2567 | 0.038 | 0.5470 | No |
| 56 | GTSE1 |  |  | 2703 | 0.033 | 0.5358 | No |
| 57 | HIST1H1C |  |  | 2712 | 0.033 | 0.5375 | No |
| 58 | DBF4 |  |  | 2751 | 0.031 | 0.5361 | No |
| 59 | MKI67 |  |  | 2760 | 0.031 | 0.5377 | No |
| 60 | FBXO5 |  |  | 2870 | 0.028 | 0.5288 | No |
| 61 | ASF1B |  |  | 2929 | 0.027 | 0.5250 | No |
| 62 | DUT |  |  | 3005 | 0.025 | 0.5193 | No |
| 63 | TMPO |  |  | 3093 | 0.023 | 0.5121 | No |
| 64 | CDK1 |  |  | 3396 | 0.016 | 0.4825 | No |
| 65 | PCLAF |  |  | 3474 | 0.014 | 0.4758 | No |
| 66 | PCNA |  |  | 3540 | 0.013 | 0.4701 | No |
| 67 | HIST1H1D |  |  | 3604 | 0.012 | 0.4646 | No |
| 68 | UHRF1 |  |  | 4092 | 0.003 | 0.4150 | No |
| 69 | SGO2 |  |  | 4184 | 0.001 | 0.4058 | No |
| 70 | NCAPG |  |  | 4261 | -0.000 | 0.3981 | No |
| 71 | MCM3 |  |  | 4633 | -0.007 | 0.3606 | No |
| 72 | CENPK |  |  | 4697 | -0.007 | 0.3548 | No |
| 73 | CHAF1A |  |  | 4943 | -0.012 | 0.3306 | No |
| 74 | MND1 |  |  | 4982 | -0.012 | 0.3277 | No |
| 75 | HIST1H1A |  |  | 5105 | -0.014 | 0.3164 | No |
| 76 | HIST1H1B |  |  | 5125 | -0.014 | 0.3155 | No |
| 77 | KPNA2 |  |  | 5461 | -0.020 | 0.2828 | No |
| 78 | MCM5 |  |  | 5588 | -0.022 | 0.2716 | No |
| 79 | CLSPN |  |  | 5848 | -0.026 | 0.2472 | No |
| 80 | RRM2 |  |  | 6214 | -0.033 | 0.2125 | No |
| 81 | E2F1 |  |  | 6247 | -0.034 | 0.2119 | No |
| 82 | SGO1 |  |  | 6841 | -0.046 | 0.1548 | No |
| 83 | CENPF |  |  | 6902 | -0.047 | 0.1524 | No |
| 84 | LMNB1 |  |  | 7166 | -0.055 | 0.1298 | No |
| 85 | EZH2 |  |  | 7260 | -0.057 | 0.1248 | No |
| 86 | MCM7 |  |  | 7397 | -0.061 | 0.1156 | No |
| 87 | RRM1 |  |  | 7556 | -0.065 | 0.1046 | No |
| 88 | MCM4 |  |  | 7658 | -0.068 | 0.0996 | No |
| 89 | TOP2A |  |  | 7678 | -0.069 | 0.1031 | No |
| 90 | CDC45 |  |  | 7751 | -0.072 | 0.1014 | No |
| 91 | RAD51AP1 |  |  | 8227 | -0.091 | 0.0599 | No |
| 92 | MCM10 |  |  | 8339 | -0.096 | 0.0561 | No |
| 93 | KIF15 |  |  | 8542 | -0.108 | 0.0439 | No |
| 94 | ESCO2 |  |  | 8566 | -0.109 | 0.0502 | No |
| 95 | SMC2 |  |  | 8721 | -0.119 | 0.0438 | No |
| 96 | HIST1H4C |  |  | 8820 | -0.126 | 0.0437 | No |
| 97 | ORC6 |  |  | 8888 | -0.133 | 0.0473 | No |
| 98 | FANCI |  |  | 8917 | -0.136 | 0.0551 | No |
| 99 | ATAD2 |  |  | 8948 | -0.138 | 0.0629 | No |
| 100 | TMSB15A |  |  | 9079 | -0.150 | 0.0614 | No |
| 101 | BRCA1 |  |  | 9600 | -0.258 | 0.0285 | No |
Table: GSEA details [plain text format]

  

Fig 2: LARSSON\_STATE\_4: Random ES distribution      
 Gene set null distribution of ES for **LARSSON\_STATE\_4**
